# Effectiveness of Intranasal Esketamine on Suicidal Ideation and Depressive Symptoms in Patients with Treatment-Resistant Depression: A Longitudinal Study

**DOI:** 10.3390/jcm15010250

**Published:** 2025-12-29

**Authors:** Matteo Leonardi, Alice Frediani, Maria Chiara Angeletti, Monica Biseo, Giada Versaci, Michele Castiglioni, Miriam Olivola, Matteo Vismara, Alberto Varinelli, Monica Bosi, Beatrice Benatti, Natascia Brondino, Bernardo Maria Dell’osso

**Affiliations:** 1 Department of Psychiatry, ASST Fatebenefratelli Sacco, University of Milan, 20157 Milan, Italy; matteo.leonardi@unimi.it (M.L.); frediani.alice@asst-fbf-sacco.it (A.F.); angeletti.maria@asst-fbf-sacco.it (M.C.A.); monica.biseo@unimi.it (M.B.); versaci.giada@gmail.com (G.V.); castiglioni.michele@asst-fbf-sacco.it (M.C.); vismara.matteo@asst-fbf-sacco.it (M.V.); varinelli.alberto@asst-fbf-sacco.it (A.V.); bosi.monica@asst-fbf-sacco.it (M.B.); dellosso.bernardo@asst-fbf-sacco.it (B.M.D.); 2 Department of Brain and Behavioral Sciences, University of Pavia, 27100 Pavia, Italy; natascia.brondino@unipv.it; 3 Department of Biomedical and Clinical Sciences, Neuroscience Research Center, University of Milan, 20157 Milan, Italy; 4 Bipolar Disorders Clinic, Department of Psychiatry and Behavioral Sciences, Stanford Medical School, Stanford University, Stanford, CA 94305, USA; 5 “Aldo Ravelli” Center for Neurotechnology and Brain Therapeutic, University of Milan, 20157 Milan, Italy

**Keywords:** esketamine, treatment-resistant depression, suicidal ideation, predictors, rapid-acting antidepressant

## Abstract

**Background**: Suicidal ideation (SI) represents a clinical challenge in patients with treatment-resistant depression (TRD), and the management of this condition should be as rapid and effective as possible. Intranasal Esketamine has shown promise in patients with TRD due to its rapid onset of action on both SI and depressive symptoms. Since this medication has been recently approved, real-world data on its efficacy remain scarce, and little is known about which patients are most likely to benefit from this approach. **Aims**: This study aimed (1) to evaluate the efficacy of intranasal Esketamine on SI and depressive symptoms and (2) to find potential predictors of clinical response. **Methods**: Patients with TRD who received intranasal Esketamine were included in this study. Clinical evaluations and psychometric assessments were made at baseline (T0) and at five subsequent time points (one week [T1], one month [T2], two months [T3], three months [T4], and six months [T5]). SI was assessed using the Columbia Suicide Severity Rating Scale (C-SSRS), and depressive symptoms were evaluated using the Montgomery–Åsberg Depression Rating Scale (MADRS). Furthermore, sociodemographic, clinical, and pharmacological data were collected. **Results**: Eighty patients diagnosed with TRD were enrolled. Suicidal ideation (C-SSRS items 1–5) decreased from 1.56 ± 1.65 at baseline to 0.78 ± 1.28 at T1 and 0.12 ± 0.52 at T5 (all *p* < 0.001). MADRS fell from 31.81 ± 7.94 to 23.62 ± 9.08 and 10.19 ± 7.33 at the same time points (all *p* < 0.001). By T1, 68.4% achieved an SI response on the C-SSRS. The MADRS response rate increased from 16.7% at T1 to an overall response of 62.5% at T5. Male sex predicted lower odds of early response on the C-SSRS (OR = 0.21, *p* = 0.031); no other baseline variable was significant as a predictor. **Conclusions**: Intranasal Esketamine has been shown to be effective in the rapid reduction and lysis of SI in patients with TRD. Male gender was found as a negative predictor of response, suggesting the importance of considering gender differences during treatment planning.

## 1. Introduction

Suicide represents one of the most significant public health challenges and remains a highly complex issue in psychiatric practice. According to the World Health Organization, more than 700,000 deaths by suicide occur worldwide each year, and approximately one in every 100 deaths is because of suicide, making it one of the leading causes of death [1]. Young adults are the ones more at risk: suicide has been identified as the second leading cause of death amongst individuals between the ages of 15–29 [1]. Notably, patients with major depressive disorder (MDD) represent a high-risk population, with a relative risk of suicide between 12 and 20 times higher than in the general population [2]. In Europe, the current overall prevalence of MDD is 6.38% [3], and about 31% of these patients have experienced lifetime suicidal ideation (SI) [4]. MDD patients with active SI have a 21-fold higher risk of committing suicide [5], and those with more severe SI (i.e., intent with a plan) are at greater risk of attempted or completed suicide [6]. SI should always be investigated in the context of MDD, since patients with SI show more severe depressive symptoms [7] and worse response to treatment [8] compared to patients without SI. Lastly, it is important to notice that patients with active SI or previous lifetime suicide attempts are less likely to improve and obtain remission with conventional antidepressants [9].

Considering this background, patients experiencing SI require prompt and comprehensive intervention to prevent self-harm [10]. The standard of care involves treatment with antidepressants, and in the most severe cases, hospitalization may be required [11]. Traditional antidepressants were known to have some degree of anti-suicidal effects, but the lack of rapid onset (about 4–6 weeks) limits their use for acute suicidal crises. Therefore, fast-acting antidepressants that specifically and rapidly reduce suicidal risk are needed for patients with severe MDD [12].

In 2019, the European Medicines Agency (EMA) approved intranasal Esketamine as an adjunctive treatment to a selective serotonin reuptake inhibitor (SSRI) or serotonin–norepinephrine reuptake inhibitor (SNRI) for adults with TRD who have failed to respond adequately to at least two oral antidepressants. This approval remains strictly as a combination therapy, so Esketamine in monotherapy is not authorized in Europe. In 2020, the Food and Drug Administration (FDA) approved Esketamine also for the treatment of depressive symptoms in adults with MDD with acute suicidal ideation or behavior in combination with an oral antidepressant.

The rapid effect of Esketamine on SI has been reported both in registration trials and “real-world” studies, where the acute effect in reducing suicidality has been assessed [13]. Real-world data on the effectiveness of Esketamine were collected and analyzed by the REAL-ESK group: the Authors ran a multicenter and retrospective study on a real-world cohort (n = 116) of patients with TRD treated with intranasal Esketamine. Depressive symptom severity, measured using the Montgomery–Åsberg Depression Rating Scale (MADRS) and the Hamilton Depression Rating Scale (HAM-D), declined significantly at 1 and 3 months; moreover, two-thirds of the patients achieved a clinical response, and about 40% reached remission at 3 months [14]. ASPIRE I and ASPIRE II were registrative, multicenter, randomized, double-blind, placebo-controlled Phase 3 trials designed to assess the rapid antidepressant and anti-suicidal effect of intranasal Esketamine (84 mg twice weekly for 4 weeks) in adults (18–64 years) with MDD and active SI with intent. Following a 24–48 h screening period, patients were randomized 1:1 to Esketamine or placebo, each administered alongside standard of care (i.e., initial hospitalization ≥ 5 days plus initiation or optimization of an oral antidepressant). The primary endpoint was the change in MADRS score 24 h after the first dose; a key secondary endpoint was the change in suicidality, as measured by the Clinical Global Impression-Severity of Suicidality-revised score (CGI-SS-r). These studies showed an improvement in the MADRS total score in patients treated with Esketamine vs. placebo at 24 h from the first administration. The same positive effect was also noted at the 4 h time point. By contrast, between-group differences in the CGI-SS-r were not statistically significant, although suicidality was reduced in both arms [15,16].

Moreover, a meta-analysis of nine randomized controlled trials (number of patients = 197) confirmed the rapid effect of Esketamine on suicidality after only one administration, showing a significant reduction in SI at 2, 4, and 24 h after the start of the treatment [17]. Although the acute effect of intranasal Esketamine on SI has been extensively reported, limited data are currently available regarding the maintenance of this effect [18]. In a recent study that included 209 patients with TRD, the reduction in SI was reported to persist for up to seven days following the last administration [18]; however, no study has yet investigated if this beneficial effect lasts after treatment discontinuation.

Since intranasal Esketamine was only recently approved, real-world data on its efficacy for SI remains scarce, and little is known about which patients are most likely to benefit. This study aimed to evaluate the effectiveness of intranasal Esketamine in reducing SI in a clinical sample of patients with TRD, treated according to the standard of care. A methodological strength of the present study is the use of the C-SSRS instead of a more subjective clinician-global measure, such as the CGI-SS-r, employed in the ASPIRE I and II studies. Using the C-SSRS, we aimed to enhance sensitivity to short-interval change, reducing measurement bias, improving reproducibility, and modeling trajectory over time. Clinical response—both SI and depressive symptoms—was examined in the short and long term, with assessments conducted immediately after the first week of administration and across subsequent follow-up times. Lastly, potential predictors of clinical response were analyzed. We hypothesized that adjunctive intranasal Esketamine could rapidly reduce SI and guarantee a sustained anti-suicidal effect during maintenance and could alleviate core depressive symptoms in parallel.

## 2. Materials and Methods

### 2.1. Patient Recruitment and Assessment

Adult patients (aged ≥18 years) were recruited from February 2021 to March 2025 at two Italian outpatient clinics: the ASST Fatebenefratelli-Sacco in Milan and the IRCCS Policlinico San Matteo in Pavia. Patients were included if they were suitable for a trial with intranasal Esketamine, according to the following judgment: patients had to fulfil the criteria for a current MDE, according to DSM-5-TR criteria [19], in the context of TRD, defined as an inadequate response to at least two antidepressants at adequate dosage, duration, and adherence. Esketamine prescription was in line with the product characteristics and the inclusion and exclusion criteria: first, in routine practice, patients were required to present with clinically persistent depressive symptomatology, despite at least two adequate antidepressant trials, and to have no history of non-response to Esketamine. Second, potential contraindications to Esketamine treatment were screened, including uncontrolled hypertension or severe cardiovascular or cerebrovascular disease, current or past psychotic disorders, substance use disorders, pregnancy, or breastfeeding. Finally, patients had to be able to attend the clinic regularly for supervised administration and the mandatory post-administration observation period and to provide written informed consent.

Clinical evaluations and psychometric assessments were conducted at different time points: baseline (T0), one week (T1), one month (T2), two months (T3), three months (T4), and six months (T5). These assessment time points were chosen to focus on both the rapid and the sustained effects of Esketamine on SI and depressive symptoms. Specifically, T1 fell within the induction phase, in which the rapid effect onset, safety, and tolerability were assessed, and T2 corresponded to the end of the induction phase, when the continuation of the treatment was evaluated based on efficacy. T3 and T4 reflected the early maintenance phase, during which dosing was usually reduced to once weekly or every two weeks. Finally, T5 was selected to explore the longer-term maintenance of clinical benefits after months of treatment. The C-SSRS is a clinician-administered scale designed to quantify the full spectrum of SI severity and related behaviors [20]. The first 5 items assess the severity of suicidality, ranging from a passive wish to be dead (1 point) to active suicidal ideation with a specific plan (5 points). These items are followed by additional items addressing preparatory behaviors and actual, interrupted, or aborted attempts. In the present study, SI severity was assessed using the C-SSRS, specifically using the sum of items 1 to 5. We decided to consider only items from 1 to 5 for the following reasons: (1) the primary outcome was the intensity of SI rather than lifetime manifestation of suicidal behavior; (2) these five items are the instrument’s core, standardized severity metric with validated points that support reliable between- and within-person comparisons [20,21]; (3) by limiting administration to the ideation subscale, we reduced participant burden and optimized assessment time within a broader battery of measures. Finally, the administration of the C-SSRS to assess suicidality is supported by other reliable studies: the studies by Greist et al. and Lindh et al. are two well-characterized and large studies that used the C-SSRS to evaluate and predict suicidality in clinical populations [21,22]. Depressive symptoms were assessed using the MADRS scale. The MADRS is a reliable and widely used ten-item clinician-rated scale designed to measure the severity, on a scale from 0 to 60, of depressive episodes in patients with mood disorders [23].

At baseline, sociodemographic data, clinical data, pharmacological data, and anamnesis were collected. At each time point, the C-SSRS and MADRS were administered by a resident psychiatrist to assess suicidal ideation and depressive symptoms. Psychometric scales were administered at each time point, not necessarily by the same operator, and to minimize inter-rater variability, all raters were experts in the diagnosis and treatment of depressive disorders and received standardized training in scale administration. Because the assessments were conducted in routine clinical practice, the operators were not formally blinded to treatment phase or to prior scores, although previous ratings were not systematically reviewed before each assessment.

To evaluate the treatment’s effect on SI, a patient was considered a responder if they showed either a reduction of at least 50% in the C-SSRS score from baseline (T0) or a score of 0 at any follow-up time point. To assess the treatment’s effect on depressive symptoms, a patient was considered a responder if they showed a reduction of at least 50% in the MADRS. Moreover, a patient was classified as an early responder if they met the response criteria at the C-SSRS by T1. Time to response was defined as the first time point at which the response criteria were met.

### 2.2. Statistical Analysis

Statistical analyses were conducted using IBM SPSS Statistics (version 27). Descriptive statistics were run for sociodemographic and clinical variables. Correlations between the C-SSRS and MADRS were evaluated at each time point using Pearson correlation coefficients. Longitudinal changes in C-SSRS scores were analyzed using linear mixed-effects models, which included time, baseline C-SSRS and MADRS scores, mean intranasal Esketamine dose, and sex as fixed effects, along with a random intercept for each patient. Mixed models were estimated using maximum likelihood and handled missing data under the assumption of missing at random; no ad hoc imputation procedures were applied. To identify predictors of early response on the C-SSRS, a logistic regression model was fitted with early response status as the dependent variable and baseline sociodemographic and clinical characteristics as independent predictors. This analysis included only patients with sufficient data to determine early response status. Statistical significance was set at *p* < 0.05.

## 3. Results

The study cohort (N = 80) had a mean age of 49.1 ± 16.8 years; 55% were women and 45% were men. Concerning marital status, 58.8% of the patients were partnered, and 41.2% were single. Overall, 46.2% of the patients were unemployed, and 53.8% were employed. Clinically, 84% of the sample experienced an MDE in the context of MDD, whereas 16% had a primary diagnosis of bipolar disorder. The mean age at onset was 31.5 ± 15.5 years, and the patients had experienced 4.8 ± 5.9 MDEs in their lifetime. The current episode had lasted a mean of 241 ± 194 days. The mean lifetime number of suicide attempts was 0.4 ± 0.8, and 70% of the patients reported a positive family history of depression or suicidal behavior. Regarding psychopharmacological treatment, 60% of the patients were receiving SSRIs, 40% SNRIs, and 43.8% other classes of antidepressants. In addition, 52.5% were treated with atypical antipsychotics (APs), 38.8% with mood stabilizers (MSs), and 56.2% with benzodiazepines (BDZs). Table 1 shows the sociodemographic and clinical variables of the sample.

**Figure 1:**
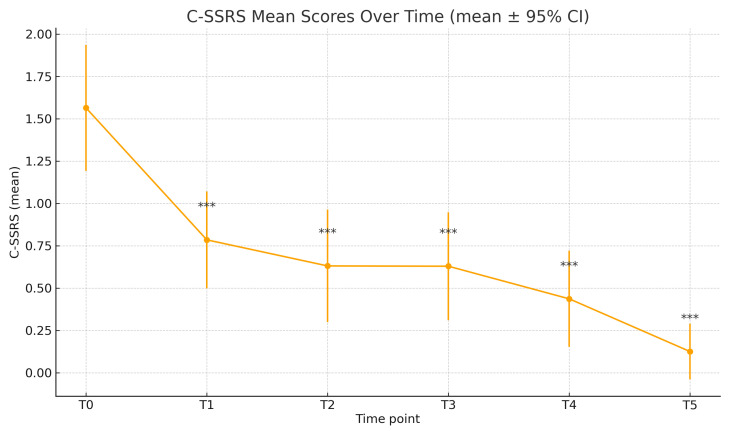
C-SSRS scores over time. *** *p* < 0.001.

**Figure 2:**
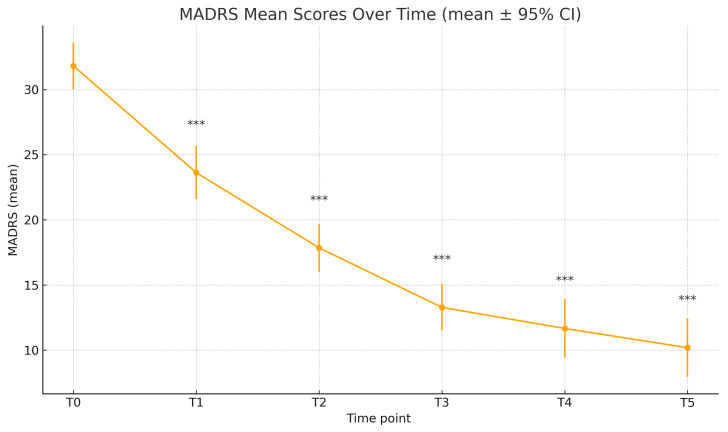
MADRS scores over time. *** *p* < 0.001.

At baseline, the mean C-SSRS score was 1.56 (±1.65), while the mean MADRS score was 31.87 (±7.94). Time progression analysis of the C-SSRS scores showed a statistically significant reduction at each time point, compared to T0 (Figure 1, Table 2); the mean C-SSRS score decreased to 0.78 ± 1.28 (T1), 0.63 ± 1.34 (T2), 0.63 ± 1.26 (T3), 0.44 ± 1.05 (T4), and 0.12 ± 0.52 (T5). Paired-sample *t*-tests confirmed significance compared to baseline for all time points (T1–T5: *p* < 0.001). Similarly, the MADRS scores decreased significantly throughout the different time points: 23.62 ± 9.08 (T1), 17.85 ± 7.83 (T2), 13.28 ± 7.13 (T3), 11.66 ± 8.37 (T4), 10.19 ± 7.33 (T5), all with *p* < 0.001 compared to T0 (Table 3, Figure 2). In this real-world cohort, there was a progressive reduction in the sample size over the six-month follow-up. Of the 80 patients assessed at baseline, the C-SSRS was completed by 79 at T1, 65 at T2, 62 at T3, 55 at T4, and 40 at T5 (Table 2); the MADRS was available for 78 patients at T1, 72 at T2, 63 at T3, 56 at T4, and 43 at T5 (Table 3).

Pearson correlation analysis conducted between the C-SSRS and MADRS showed moderate and statistically significant correlations in the first five time points: T0 (r = 292, *p* = 0.009), T1 (r = 313, *p* = 0.005), T2 (r = 0.36, *p* = 0.003), T3 (r = 0.295, *p* = 0.02), and T4 (r = 0.317, *p* = 0.018). At T5, the correlation was weaker (r = 0.189) and not significant (*p* = 0.244).

**Figure 3:**
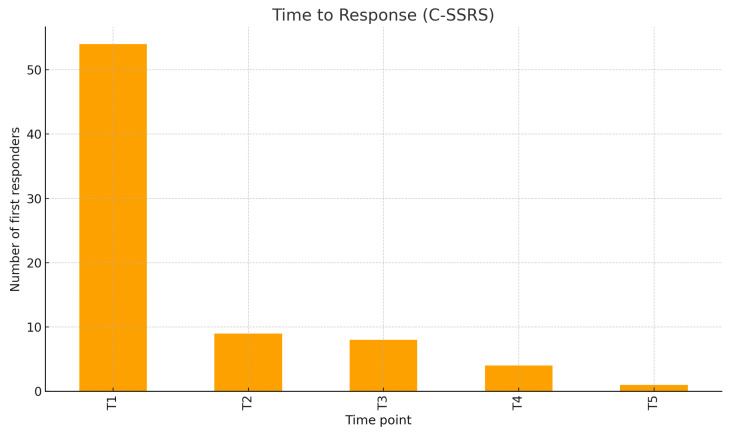
Distribution of clinical response over time (C-SSRS).

**Figure 4:**
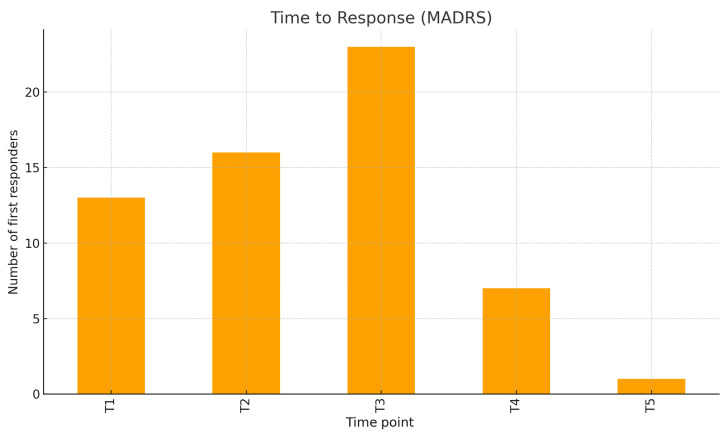
Distribution of clinical response over time (MADRS).

The C-SSRS clinical definition for response (≥50% reduction or final score 0) was reached by 97.5% of the patients evaluated at T5. The time to response (considering the C-SSRS) peaked at T1 (54 patients, 68.35%), with fewer first responses at T2–T5: respectively, 9, 8, 4, and 1 (Figure 3). Considering the MADRS, the response rates increased from 16.7% at T1 to 37.5% at T2, 69.8% at T3, 76.8% at T4, and 76.7% at T5, with an overall MADRS response rate of 62.5. The distribution of time to response on the MADRS is shown in Figure 4.

Logistic regression showed that male gender was significantly associated with a lower likelihood of early response on the C-SSRS (OR = 0.212, *p* = 0.031). The lifetime number of SAs showed a negative—but non-significant—association with early C-SSRS response at T1 (OR = 0.54, *p* = 0.143). No other variables, such as age, diagnosis, or medications at T0, showed statistical significance.

## 4. Discussion

Data from this longitudinal study confirmed and reinforced the efficacy of intranasal Esketamine in rapidly reducing SI in patients with TRD. Seventy-four percent of responders achieved the response criteria on the C-SSRS within the first week, and this improvement was maintained at six-month follow-up. The results that emerged in this paper confirm those of the ASPIRE I and II trials, which demonstrated a clinically and statistically significant reduction within 24 h after the administration of intranasal Esketamine, compared to a placebo. Previous studies, including ASPIRE I and II, used the specific item on suicide in the MADRS as a variable to measure suicidality, relying on indirect measures of SI. This study incorporated the C-SSRS, offering a more robust and specific evaluation of SI. A key strength of the present study is the use of the C-SSRS as the primary endpoint to quantify SI with a validated instrument. Many previous trials on Esketamine prioritized depressive symptom change, using the MADRS or the HAM-D, and tracked suicidality using the C-SSRS only as a secondary or safety outcome [24,25,26]. By elevating the C-SSRS as a primary outcome, our design directly addresses anti-suicidal effects rather than inferring them from depression measures.

Furthermore, the observed correlation between the MADRS and C-SSRS scores in the early phases of treatment and their progressive reduction over time may suggest a partial distinction between improvements in depressive symptoms and SI. This interesting finding supports the hypothesis that Esketamine may exert anti-suicidal effects via mechanisms that could be, at least partially, different from its antidepressant action [27]. Exploratory analyses have identified the baseline severity of SI and depressive symptoms as potential predictors of a more rapid and stronger treatment response. These findings are similar to previous data published in the literature: patients characterized by greater baseline severity of illness typically exhibit a greater magnitude of response [28]. From a clinical perspective, this suggests that more severely ill patients may benefit from early intervention with Esketamine. The rapid reduction in SI observed within the first week of Esketamine treatment underscores the crucial need for prompt therapeutic interventions in patients with TRD presenting with active SI. In this population, the temporal dimension of treatment efficacy is not merely a matter of response, but of survival. These findings reinforce the unique role of Esketamine as an emergency intervention in affective crises, distinct from traditional antidepressants both in mechanism and latency of effect.

Interestingly, male gender was found to be a possible negative predictor of early response measured on the C-SSRS. Prior studies have explored gender differences in response to antidepressants in general [29]. Biological factors, such as sex hormones influencing glutamatergic transmission, differences in brain structure, or pharmacokinetic variability, may partially account for this finding [30]. Several pharmacodynamic and pharmacokinetic mechanisms may contribute to the sex-specific response to Esketamine: neurosteroids—such as estrogens and progesterone—modulate glutamatergic and GABAergic transmission and synaptic plasticity and exert sexually dimorphic effects on brain function, providing a biological basis for differential neuroplastic responses to glutamatergic agents in women [31]. Moreover, Ketamine—and so its isomer Esketamine—are metabolized by different polymorphic cytochrome P450 enzymes (i.e., CYP2B6, CYP3A4, CYP2C9, and CYP2A6), whose activity is influenced by genetic variants, medications, and sex- and hormone-related factors, leading to interindividual variability in plasma and brain exposure for the same administration dose [32]. Although consistent and robust sex differences in the antidepressant efficacy or tolerability of Ketamine were not found, the majority of available studies were underpowered or did not run sex-stratified analyses [33]. Notably, real-world data on intranasal Esketamine have recently documented gender differences in trajectories of suicidality and self-harming behavior over treatment [34], supporting the hypothesis that these biological mechanisms, in combination with sociocultural factors, may help explain the lower probability of early anti-suicidal response observed in men in our sample.

Sociocultural factors, including differential help-seeking behaviors and stigma, may also play a role. Sociocultural factors rooted in male traditional culture, such as self-reliance, emotional stoicism, and fear of being perceived as weak, can lead men to delay help-seeking for depressive symptoms. Moreover, elevated levels of self-stigma and negative attitudes toward psychological care reduce honest reporting of SI at baseline and delay engagement in pharmacological and/or psychological treatment. Consequently, a large part of the male population starts evidence-based treatment only at crisis points, shortening the time for detecting rapid improvements and early response [34,35]. Future studies should prioritize sex-stratified analyses to better understand these dynamics and move toward more personalized treatment approaches. If confirmed, these findings could inform sex-sensitive treatment planning and contribute to more personalized approaches in psychiatric care.

The fact that no other baseline sociodemographic or clinical variable emerged as a significant predictor must be interpreted with caution: given the small sample size, our study was powered to detect only medium-to-large effects. Smaller associations may have gone undetected, and the positive finding for sex remains exploratory. Future studies with larger samples are needed to confirm if male sex is a robust negative predictor and which other variables could be predictors as well.

Although the number of previous suicide attempts was not a statistically significant predictor of final response, the observed trend was clinically interesting. As reported in the literature, individuals with a history of suicidal behavior may exhibit diminished responsiveness to antidepressants in general [36]. Larger studies are needed to determine if a positive history of suicide attempts is a reliable predictor of Esketamine efficacy. If this trend is confirmed, it would strengthen the rationale for early intervention with Esketamine before recurrent suicidal crises further complicate clinical outcomes.

For a correct interpretation of the present findings, the present limitations must be acknowledged. First, the limited sample size and exploratory nature of the analyses limit the generalizability of the findings. Second, the observational design and the lack of a placebo preclude causal inference, and unmeasured confounding factors may have influenced the outcomes. Third, although the C-SSRS provides a more nuanced evaluation of suicidality, other aspects, such as impulsivity, were not systematically assessed and could modulate treatment response.

## 5. Conclusions

In conclusion, intranasal Esketamine represented a promising option for the treatment of suicidality and depressive symptoms in patients with TRD. Future research should focus on predictor stratification, such as robust identification of clinical, demographic, and biological predictors of treatment response to guide personalized interventions. Integration of predictive modeling techniques, including machine learning algorithms applied to multimodal data (e.g., clinical, genetic, imaging, and electrophysiological markers), could pave the way for the development of validated, clinically actionable predictive algorithms. Trials designed specifically to assess sex differences and interventions tailored to patients with a history of suicide attempts represent additional priorities for future research.

## Figures and Tables

**Table 1:** Sociodemographic and clinical variables of the sample.

Variables	
**Mean age (±SD)**	49.1 ± 16.8 years
**Gender**	55% female 45% male
**Relationship status**	58.8% partnered 41.2% single
**Employment status**	46.2% unemployed 53.8% employed
**Diagnosis**	84% MDE in MDD 16% MDE in BD
**Age at first MDE (mean ± SD)**	31.5 ± 15.5 years
**Lifetime number of MDE**	4.8 ± 5.9
**Current MDE duration (mean in days ± SD)**	241 ± 194 days
**Lifetime number of SAs (mean ± SD)**	0.4 ± 0.8
**Family history for depression or SAs (%)**	Yes 70% No 30%
**Psychopharmacotherapy at baseline**	
**SSRIs**	60%
**SNRIs**	40%
**Other ADs**	43.8%
**Atypical APs**	52.5%
**MSs**	38.8%
**BDZs**	56.2%

ADs: antidepressants; APs: antipsychotics; BD: bipolar disorder; BDZs: benzodiazepines; MDD: major depressive disorder; MDE: major depressive episode; MSs: mood stabilizers; SAs: suicide attempts; SD: standard deviation; SNRIs: serotonin and noradrenalin reuptake inhibitors; SSRIs: selective serotonin reuptake inhibitors.

**Table 2:** C-SSRS scores at different time point.

	N CSSRS	C-SSRS ± SD	*p*	Cohen’s d
T0	80	1.56 ± 1.65	—	
T1	79	0.78 ± 1.28	<0.001	0.99
T2	65	0.63 ± 1.34	<0.001	1.58
T3	62	0.63 ± 1.26	<0.001	2.11
T4	55	0.44 ± 1.05	<0.001	2.03
T5	40	0.12 ± 0.52	<0.001	1.82

C-SSRS: Columbia Suicide Severity Rating Scale; N: number of patients; SD: standard deviation.

**Table 3:** MADRS scores at different Time point.

	N MADRS	MADRS ± SD	*p*	Cohen’s d
T0		31.81 ± 7.94	—	
T1	78	23.62 ± 9.08	<0.001	0.59
T2	72	17.85 ± 7.83	<0.001	0.67
T3	63	13.28 ± 7.13	<0.001	0.58
T4	56	11.66 ± 8.37	<0.001	0.76
T5	43	10.19 ± 7.33	<0.001	1.17

MADRS: Montgomery–Åsberg Depression Rating Scale; N: number of patients; SD: standard deviation.

## Data Availability

Data are unavailable due to privacy.

## Abbreviations

The following abbreviations are used in this manuscript:

AD	Antidepressant
AP	Antipsychotic
ASST	Azienda Socio-Sanitaria Territoriale
BD	Bipolar Disorder
BDZ	Benzodiazepine
C-SSRS	Columbia–Suicide Severity Rating Scale
CGI-SS-r	Clinical Global Impression-Severity of Suicidality-revised
DSM-5-TR	Diagnostic and Statistical Manual of Mental Disorders, 5ª ed., Text Revision
EMA	European Medicines Agency
FDA	Food and Drug Administration.
GCP	Good Clinical Practice
HAM-D	Hamilton Depression Rating Scale
IRCCS	Istituto di Ricovero e Cura a Carattere Scientifico
MADRS	Montgomery–Åsberg Depression Rating Scale
MDD	Major Depressive Disorder
MDE	Major Depressive Episode
MS	Mood Stabilizer
OR	Odds Ratio
SA	Suicide Attempt
SD	Standard Deviation
SNRI	Serotonin–Norepinephrine Reuptake Inhibitor
SSRI	Selective Serotonin Reuptake Inhibitor
SI	Suicidal Ideation
TRD	Treatment-Resistant Depression
